# Cannabinoid-Associated Hyperemesis Syndrome Treated With Dronabinol: Killing a Poison With the Poison

**DOI:** 10.7759/cureus.49629

**Published:** 2023-11-29

**Authors:** Azhar Hussain, Sistu KC, FNU Sapna

**Affiliations:** 1 Internal Medicine, State University of New York Upstate Medical University, Syracuse, USA; 2 Medicine, Patan Academy of Health Sciences, Kathmandu, NPL

**Keywords:** cannabis legalization, cyclic hyperemesis syndrome (chs), chronic cannabis use, dronabinol, cannabinoid hyperemesis syndrome

## Abstract

Cannabinoid hyperemesis syndrome (CHS) is a medical condition characterized by recurrent nausea, vomiting, and abdominal pain in individuals who frequently use cannabis. This case report highlights the successful treatment of CHS using dronabinol, a synthetic cannabinoid compound. A 21-year-old female presented with severe abdominal symptoms, including vomiting and pain, alongside a history of chronic cannabis use. Despite initial symptomatic treatment, her symptoms persisted. After being diagnosed with CHS, the patient was administered one dose of haloperidol, which led to agitation and worsening of her symptoms. Eventually, she was given one dose of dronabinol resulting in significant symptom improvement. Subsequent doses of dronabinol led to the complete resolution of her CHS symptoms. This case underscores the importance of thorough history-taking, especially for complex patients. Also, with cannabis legalization, cases of CHS are on the rise, and widespread awareness is vital for healthcare practitioners to recognize and appropriately manage nausea and vomiting induced by long-term cannabis intake. Although this case provides valuable insights, its limitations emphasize the need for further research to establish evidence-based guidelines for CHS management.

## Introduction

Cannabinoid hyperemesis syndrome (CHS) is characterized by recurring bouts of nausea, vomiting, and severe abdominal pain in individuals who frequently use cannabis [1,2]. The prevalence of CHS is on the rise, especially after the legalization of cannabis use in the United States, and is reported to be 0.1%-35% in the setting of chronic cannabis use [3]. With 74% of Americans having access to some form of marijuana, this $35 billion industry made 12% of Americans smoke marijuana, with 44% of young college students in 2022. Although the exact cause of CHS is not fully understood, it is suspected that it involves activating cannabinoid receptors in the digestive tract due to chronic use [1,4]. Before confirming CHS, other potential causes of recurring vomiting, such as gastrointestinal (GI) blockages or metabolic disorders, must be ruled out. Unlike other forms of cyclic vomiting syndrome (CVS), CHS patients typically lack a history of migraines or other psychological stressors [5,6]. Moreover, an exclusive feature of CHS involves the compulsion to take frequent hot showers or baths to alleviate symptoms, a behavior distinct from other CVS variants [7].

Dronabinol, a synthetic cannabinoid compound, has been employed to treat CHS, but with mixed efficacy and safety profile. Its effects include slowing stomach emptying, suppressing colon contractions, and causing loss of colon tone by binding to CB1 receptors in nerve fibers of the gut5. Although dronabinol remains a questionable compound for the management of CHS, some case reports demonstrated it to be effective in alleviating symptoms and preventing further vomiting episodes in CHS patients [7,8]. This case report emphasizes the safe and effective use of dronabinol as a viable treatment for CHS in a young female.

## Case presentation

A 21-year-old female, with an insignificant past medical history, presented with intermittent and cyclical vomiting and generalized abdominal pain for two weeks. She had been having diarrhea and on-and-off constipation for the past 10 months. She has been spending four hours a day on the toilet. Abdominal pain is severe, colicky, and cyclical associated with vomiting and relieved with hot showers or hot tub baths and not relieved with acetaminophen or nonsteroidal anti-inflammatory drugs (NSAIDs). Her pain was severe and excruciating to the point of making her cry. Professionally, she was a hairdresser and had to cancel many appointments because of the pain. Her home medications include omeprazole and Tums as needed. Her last menstrual period was 2-3 weeks before admission. Over the past 24 hours of admission, she had reported vomiting around 20 times. Initially, vomiting was projectile and contained gastric content, and then, it became bilious.

In the emergency department (ED), her vitals were blood pressure of 135/86 mmHg, pulse of 96 beats/minute, respiratory rate of 22 breaths/minute, and temperature of 36°C. An electrocardiogram (EKG) showed sinus rhythm. Laboratory results noted glucose of 185 mg/dL (normal: 70-99 mg/dL), creatinine of 1.06 mg/dL (normal: 0.6-1.2 mg/dL), aspartate aminotransferase (AST) of 44 U/L (normal: <40 U/L), alanine aminotransferase (ALT) of 75 U/L (normal: <40 U/L), lipase of 18 U/L, and high-sensitivity troponin I of 6 ng/L (normal: <14 ng/L). Complete blood count (CBC) with differential revealed white blood cell (WBC) of 18.2 × 10^3^/µL (normal: 4.5-11 × 10^3^/µL) and hemoglobin of 14 g/dL (normal: 12-16 g/dL) (Table 1). Urinalysis was unremarkable with a specific gravity of 1.043. Computed tomography (CT) of the abdomen and pelvis with contrast were also unremarkable.

**Table 1:** Laboratory parameters of the patient

Parameter (unit)	Laboratory value	Reference range
White cell count, total (× 10^3^/µL)	18.2	4.5-11
Hemoglobin, serum g/dL	14	12-16
Glucose, serum (mg/dL)	185	70-99
Creatinine, serum (mg/dL)	1.06	0.6-1.2
Aspartate aminotransferase (U/L)	44	<40
Alanine aminotransferase (U/L)	75	<40
Lipase, serum (U/L)	18	0-160
High-sensitivity troponin I (ng/L)	6	<14

In the ED, she received fentanyl 25 mcg IV, prochlorperazine 10 mg twice, and ondansetron 4 mg IV, without significant improvement in her symptoms. She also received a 1 L bolus of normal saline. Eventually, she was given lorazepam 2 mg, which led to improvement in her condition. During the whole hospital stay, her vitals remained stable.

Gastroenterology (GI) was consulted to evaluate and treat her condition. GI recommended esophagogastroduodenoscopy (EGD) for peptic ulcer disease. EGD revealed mild gastritis and some evidence of reflux esophagitis without bleeding. Patchy mild inflammation characterized by congestion (edema) and erythema was found in the prepyloric region of the stomach. Biopsies were taken with cold forceps for histology. Cold snare biopsies were taken from the gastric antrum and duodenum, which came negative for *Helicobacter pylori*. Esophageal biopsy indicated mild reflux esophagitis without dysplasia. GI recommended pantoprazole 40 mg IV twice daily on admission to be continued upon discharge as well. Subsequently, her symptoms improved, and she was tolerating her diet from clear liquids to a solid diet. After four days of hospitalization, she was discharged with acetaminophen, aluminum, and magnesium hydroxide-simethicone 200-200-20 mg/mL oral suspension, capsaicin 0.1 % topical cream, cyclobenzaprine 10 mg tablet, pantoprazole 40 mg capsule, and simethicone 80 mg chewable tablet.

Only about 1.5 hours after hospital discharge, she landed in the ED again with the same intractable abdominal pain and four episodes of non-bloody vomiting.

While she had evidence of mild gastritis and mild reflux esophagitis on her endoscopy, recurrent and severe symptoms were not consistent with the severity of direct endoscopic visualization of the stomach and distal esophagus. Due to the severity of her disabling symptoms, an extensive history was taken by the medical team, which revealed a nine-month-long use of two different forms of marijuana, i.e., smoking weed and a marijuana pipe. In the absence of other organic etiologies for her presentation, she met the Diagnostic and Statistical Manual of Mental Disorders, Fifth Edition (DSM-5) criteria for cannabinoid-associated hyperemesis syndrome with intractable abdominal pain and cyclic vomiting due to marijuana use. During the second hospitalization, the patient was administered one dose of haloperidol, which led to agitation and worsening of her symptoms. Subsequently, she was given one dose of dronabinol 2.5 mg, dramatically improving her abdominal pain from 10/10 to 4/10, as well as vomiting to barely any. After the third dose of dronabinol, she reported complete resolution of abdominal pain, nausea, and vomiting. Prior to discharge, the patient was educated about her diagnosis of marijuana-induced cyclic vomiting syndrome and marijuana use in different forms as the primary culprit of her suffering. She was strongly advised against the use of marijuana. The patient affirmed compliance to complete abstinence from marijuana of any form. She was discharged home with a three-week supply of dronabinol 2.5 mg per oral twice daily and metoclopramide as needed, with outpatient follow-up with her primary care provider (PCP).

Upon the third week of post-discharge follow-up, she had recovered from all CHS symptoms and was off dronabinol. She was also abstaining completely from all forms of marijuana or relevant derived products. Also, she denied any forms of cravings for marijuana. She also joined her gym and started going to her job.

## Discussion

Cannabinoid hyperemesis syndrome (CHS) is defined by repeated cycles of nausea and vomiting in people who frequently use cannabis. The prevalence of CHS in the available literature spans from 0.1% to 35% in chronic cannabis users [3]. It is more commonly observed in males who use cannabis every day or at least once a week and have used cannabis for over a year [2]. CHS often goes undiagnosed because it resembles other illnesses and there is limited research on it. A case report published by Bellamy et al. (2023) [4] describes the case of a 42-year-old male with extensive long-term cannabis use who displayed symptoms typical of CHS. The likelihood of the hyperemesis being linked to cannabinoid use was assessed using the Naranjo adverse drug reaction probability scale, resulting in a probable score that strongly connected CHS with cannabis use [9]. However, the exact mechanism behind CHS is not fully understood. It is thought to involve problems with the endocannabinoid system, leading to unusual movements in the digestive system [4].

**Figure 1:**
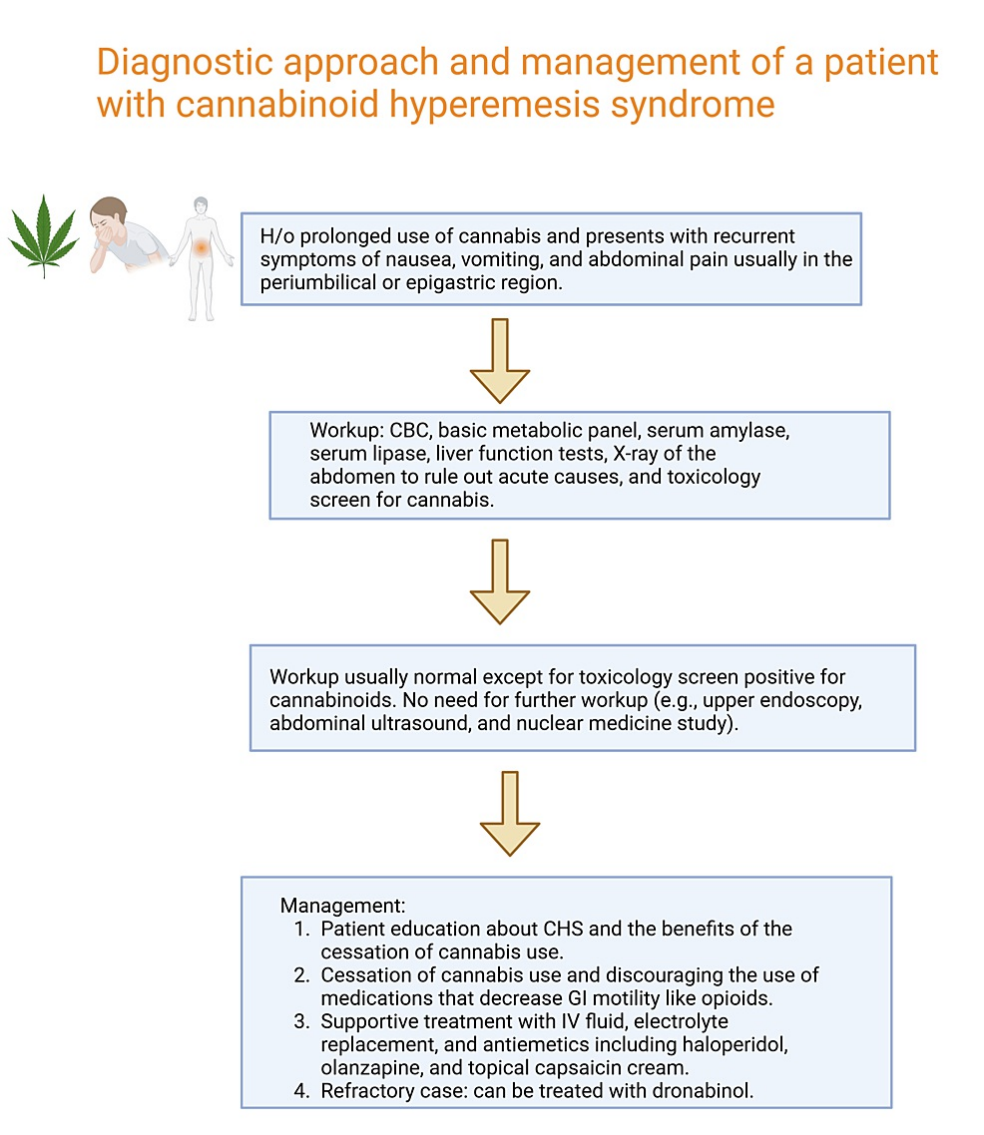
Diagnostic approach and management of cannabinoid hyperemesis syndrome Figure 1 is designed and drafted by the authors. CBC: complete blood count, CHS: cannabinoid hyperemesis syndrome, GI: gastrointestinal, IV: intravenous

The first line of treatment for CHS entails discontinuing cannabis use and providing supportive measures, including hydration and antiemetic medications [2]. Also, patients with CHS often have a habit of taking hot baths or showers, which they have learned helps them feel better temporarily [10-12]. However, why exactly this works is not clear [11,12]. These compulsive showers or baths seem to help lessen CHS symptoms [12]. The second-line agents include haloperidol, olanzapine, or topical capsaicin. Although some case reports have reported good outcomes in CHS with triptans (sumatriptan), we need more randomized controlled trials (RCTs) based on data on the triptans' efficacy and long-term safety in marijuana-induced CHS. Dronabinol, which is a synthetic of delta-9-tetrahydrocannabinol (THC), has been used to manage nausea and vomiting caused by chemotherapy [10]. It has been shown to be just as effective, if not better, than regular anti-nausea drugs [10]. In a report about a pregnant patient with CHS, they found relief from symptoms using diphenhydramine, haloperidol, and dronabinol [6]. However, the use of dronabinol for CHS is not widely studied, as most studies have focused on its use for chemotherapy-related nausea and vomiting [11]. Dronabinol, a compound that activates various cannabinoid receptors, has been proven to slow down stomach emptying and decrease the tone and rhythmic contractions of the colon [4,9,13], which could explain its efficacy in relieving CHS symptoms in the setting of chronic cannabis use. Figure 1 shows the suggested workup and management of cannabinoid hyperemesis syndrome (CHS).

One limitation lies in its reliance on a solitary case without a comparison group, which hampers the applicability of the findings. Despite our case offering evidence of successful dronabinol treatment, it is important to acknowledge that responses to treatment can differ among individuals. Further research is necessary to determine the efficacy of diverse treatment choices for CHS and to uncover factors that could forecast treatment responses. Randomized controlled trials are imperative to evaluate the efficacy of varied treatment options for CHS and to formulate evidence-based guidelines for its management.

Despite limitations, this report emphasizes the safety and efficacy of dronabinol in patients with recurrent CHS symptoms, including nausea and vomiting due to chronic cannabis use. Moreover, the report emphasizes the underdiagnosis of CHS due to its resemblance to other conditions and limited research in the field.

## Conclusions

With the increasing prevalence of cannabis in the United States and worldwide, our case highlights the need for consideration of CHS as a differential diagnosis in the setting of chronic cannabis use. With the worldwide growing usage of cannabis due to its legalization, cases of CHS are on the rise, and widespread awareness is vital for healthcare practitioners to recognize and appropriately manage nausea and vomiting induced by long-term cannabis intake.
